# Differential Network Analysis Reveals Genetic Effects on Catalepsy Modules

**DOI:** 10.1371/journal.pone.0058951

**Published:** 2013-03-21

**Authors:** Ovidiu D. Iancu, Denesa Oberbeck, Priscila Darakjian, Sunita Kawane, Jason Erk, Shannon McWeeney, Robert Hitzemann

**Affiliations:** 1 Department of Behavioral Neuroscience, Oregon Health & Science University, Portland, Oregon, United States of America; 2 Oregon Clinical and Translational Research Institute, Oregon Health & Science University, Portland, Oregon, United States of America; 3 Division of Biostatistics, Public Health & Preventative Medicine, Oregon Health & Science University, Portland, Oregon, United States of America; 4 Research Service, Veterans Affairs Medical Center, Portland, Oregon, United States of America; CRS4, Italy

## Abstract

We performed short-term bi-directional selective breeding for haloperidol-induced catalepsy, starting from three mouse populations of increasingly complex genetic structure: an F_2_ intercross, a heterogeneous stock (HS) formed by crossing four inbred strains (HS4) and a heterogeneous stock (HS-CC) formed from the inbred strain founders of the Collaborative Cross (CC). All three selections were successful, with large differences in haloperidol response emerging within three generations. Using a custom differential network analysis procedure, we found that gene coexpression patterns changed significantly; importantly, a number of these changes were concordant across genetic backgrounds. In contrast, absolute gene-expression changes were modest and not concordant across genetic backgrounds, in spite of the large and similar phenotypic differences. By inferring strain contributions from the parental lines, we are able to identify significant differences in allelic content between the selected lines concurrent with large changes in transcript connectivity. Importantly, this observation implies that genetic polymorphisms can affect transcript and module connectivity without large changes in absolute expression levels. We conclude that, in this case, selective breeding acts at the subnetwork level, with the same modules but not the same transcripts affected across the three selections.

## Introduction

Haloperidol is a member of a class of drugs denoted as “typical” or first-generation antipshychotics since their adoption as psychosis treatments. A number of locomotory side effects, including dyskinesia, complicate their use in humans. Mice injected with haloperidol develop catalepsy (rigid, fixed body position) and this response is considered a model for human locomotory disturbances. Mouse inbred strains differ widely in their response to haloperidol, signaling a strong genetic component for this trait; a number of associated genetic loci have been previously detected in mouse crosses [1]. Additionally, a number of studies implicate the striatal dopaminergic system in the cataleptic response [2], [3].

We have examined the striatal gene-expression covariance structure in animals that were selectively bred from a heterogeneous stock (HS) for haloperidol response and non-response [4]. Crossing the C57BL/6J (B6), DBA/2J (D2), BALB/cJ (C) and LP/J (LP) inbred mouse strains formed the HS founder population. This HS cross, denoted as HS4, is of intermediate genetic complexity [5]. After three generations of selection, the responsive and non-responsive lines differed more than 30-fold in the haloperidol-induced catalepsy ED_50_. The lines also differed in their response to structurally dissimilar D_2_ dopamine receptor antagonist, raclopride, strongly suggesting that the basis for selection was pharmacodynamic and not pharmacokinetic. Selection produced only modest changes in striatal gene expression despite the use of large populations (N =  >40/line). In contrast, applying Weighted Gene Coexpression Network Analysis (WGCNA) [6] to the microarray data revealed significant changes in gene connectivity, most notably in a module enriched in genes associated with cell signaling and behavior. Previous studies have highlighted the genetic influences on trait variability, which can be distinct from influence on average trait values [7]. Gene coexpression networks are constructed on the basis of gene or transcript joint variability; genetic effects on variability will therefore translate into changes in the coexpression network structure. The adoption of graph/network techniques and terminology to the analysis of high-throughput biological data offers a distinct advantage: highly dimensional interactions can be efficiently summarized and related to specific biological or disease states. These approaches have been successfully applied in several recent studies [4], [8], [9], [10], [11]. There is, however, a potential confound in applying these techniques to data generated from selectively bred animals. Selection gives rise to numerous genetic loci that segregate between the lines; genetic drift and random allele fixation confound the functional interpretation of these loci, including their downstream effects on network connectivity. One strategy for dealing with genetic drift is to have at least a second independent selection and determine if the same association appears [12]. If the association is simply stochastic, replication is highly unlikely. In the current study, we employed a somewhat different strategy. In addition to independent catalepsy selections, we have also varied the genetic background. Two different founder populations were used; one was an F_2_ intercross formed from the B6 and D2 strains, and the other was the HS-CC that was formed from the eight founder strains of the Collaborative Cross (CC). Using single nucleotide polymorphisms (SNPs) as a surrogate for genetic diversity, the F_2_ intercross is 50% less diverse than the HS4, and the HS-CC is three times more diverse [5]. Previously, we have shown [13] that striatal network structure in the three founder populations displays significant preservation; here, we evaluate whether selection produces changes in gene coexpression independent of genetic background. The focus is on detection and statistical evaluation of changes in coexpression patterns, which we denote as module disruption. It is important to note that module preservation and module disruption are related and complementary concepts and they can both hold for a given module. Even though modules might be highly preserved across biological conditions, this does not preclude the emergence of subtle changes in network structure that are not enough to render the module non-preserved, but nevertheless are statistically significant and, potentially, biologically meaningful. To detect these changes, we adapted the module preservation procedures outlined in [14]. To examine this issue, the key strategy was the creation of a consensus gene network, using the gene expression data from all three selections.

## Materials and Methods

### Ethics Statement

All animal care, breeding and testing procedures were approved by the Laboratory Animal Users Committees at the Veterans Affairs Medical Center, Portland, OR, and at the Oregon Health & Science University, Portland, OR.

### Animal breeding and selection

The formation of the HS colonies was described previously [15]. Haloperidol response phenotyping followed the procedure outlined in [1], [4]. On day 1, all animals were administered 4 mg/kg intraperitoneally of haloperidol, and the catalepsy response, which consists of remaining in a fixed rearing posture for 30 seconds, was measured after 15 minutes. Based on this response, the animals were categorized as haloperidol responders (R) and non-responders (NR). After a week, the R animals were administered 1 mg/kg of haloperidol, and the NR animals were administered 7 mg/kg of haloperidol; the second test produced two additional categories: very-responsive and very non-responsive animals. Breeding sixteen families of very-responsive and very-nonresponsive animals produced the first selection generation; three additional breeding generations resulted in the High (responsive) and the Low (non-responsive) selected lines. Additional details and the general strategy for short-term selective breeding is found in [12].

### Gene-expression data processing

Our data pre-processing steps closely follow procedures described previously [13]. Gene-expression data for the High and Low selected lines were obtained from the striatum using the Illumina WG 8.2 array exactly as described by the manufacturer. The dissection of the striatum and the details of sample preparation for hybridization are found in [15]. Data were imported into the R application environment (http://www.r-project.org), and outlier samples were removed. Probes not expressed above background (p<0.01) in at least a quarter of the samples were removed. For each probe, we computed the coefficient of variability (CV) and we selected for network construction the top 50% most variable probes. The intersection of these probes across the three datasets resulted in 6755 probes. The data were quantile normalized. Microarray datasets are publicly available in the Gene Expression Omnibus database [16] under accession number GSE37755.

### Quantification of genetic variability and genetic differences

F_2_ and HS4 selected lines were genotyped using a panel of 768 SNPs previously described [15]. The HS-CC samples were genotyped using the Mouse Universal Genotyping Array, which uses 7851 SNP markers distributed over the mouse genome (http://www.neogen.com/GeneSeek/SNP_Illumina.html). Genome-wide genetic differences were analyzed following the AMOVA procedure [17]. Each genome was encoded as a long vector with entries of 0, 1 or 2 reflecting marker allelic content; pairwise “manhattan” distances were computed between these vectors. Subsequently, between versus within groups (High or Low selections) distances were evaluated in a manner similar to the classic analysis of variance approach. The genome-wide pairwise distances were visualized using the multidimensional scaling procedure available in the R application environment.

### Coexpression network construction and validation

We constructed the coexpression network using the WGCNA approach [6], [18]. First, the absolute value of the Pearson correlation coefficient between all transcript pairs across samples was computed, resulting in a correlation matrix. The Pearson correlation matrix was subsequently transformed into an adjacency matrix (A) using a power function. The connection strength a_ij_ between transcripts x_i_ and x_j_ then becomes a_ij_ = |corr(x_i,_ x_j_ )|^β^; β = 10 was selected in accordance to the scale-free topology criterion [6]. To detect modules or groups of coexpressed transcripts, the adjacency matrix was clustered using the “dynamic tree cut” algorithm [19]; this procedure takes advantage of the internal structure of the dendrogram in cutting the branches and identifying modules. We validated module membership by a permutation procedure, checking whether average module adjacency is higher than average adjacency of random groups of transcripts. For the detection of TFBSs in the upstream regions of module genes, we used the Promoter Analysis and Interaction Network Tool (PAINT) [20]; once the TFBSs for each gene were collected we used Fisher’s exact test followed by FDR adjustment to test for overrepresentation of each module TFBS against the whole network.

### Differential network analysis and detection of module disruption

Recent work [14] has introduced a comprehensive and powerful method for evaluation of preservation of network properties. For any collection of network nodes of interest (module), preservation statistics are created by comparing network/module statistics against similar values compiled from randomly selected groups of nodes. Broadly speaking, bootstrapping or random selection is performed over network nodes. In the current application, the goal is to detect significant changes in network structure. To detect these changes, we adapted the module preservation procedures outlined above. In essence, we create separate networks corresponding to the two biological conditions; differences between these networks are evaluated against changes that could occur by chance. An empirical distribution of random changes was generated by constructing a set of networks (N = 200) using a mixture of samples from both biological conditions. Bootstrapping was performed over samples as opposed to network nodes. This procedure is feasible in our dataset because the samples corresponding to the High and Low lines originate from the same tissue and were processed together as part of the same experiment. We computed network preservation statistics, exactly as defined in [14], Equations 1–20, for pairs of networks from this empirical distribution. The network statistics used here include intramodular connectivity (kIM), total network eigengene connectivity (kMEAll), module eigengene connectivity (kME), clustering coefficient (clusterCoeff) and maximum adjacency ratio (MAR). These quantities apply to individual nodes; for a given module the values for all nodes are arranged in a vector. Vectors originating from two different networks are correlated, resulting in cor.kIM, cor.kMEAll, cor.kME, cor.clusterCoeff and cor.MAR; cor.ADJ is obtained from matrix correlation of the adjacency matrices. High correlation values correspond to strong preservation. Furthermore, the statistical significance of preservation or disruption values can be quantified using a Z score [14]: 
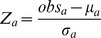
, where *obs_a_* corresponds to preservation for a given network statistic and module, μ_a_ and σ_a_ correspond to mean and standard deviation of preservation values, in our case generated from the empirical distribution of preservation values between mixed-sample networks. While for module preservation the Z scores are positive, signifying that modules are more preserved than random groups of nodes, disruption Z scores are often negative. The negative Z values result from the fact that preservation between two networks corresponding to different biological conditions is lower than preservation between two networks created using a mixture of samples: often *obs_a_* is smaller than μ_a_. Following [14], we consider Z scores lower than -2 disrupted.

### Inference of ancestral allele origin and allelic imbalance

The HS-CC dense genotype data, which included genotypes of the eight parental strains, was used to infer the probability of each ancestral allele for each combination of genomic interval and each individual. We used a dynamic programming algorithm available as part the R package HAPPY [21]. The alleles with highest probability for each sample/interval were arranged in a contingency table and imbalance was evaluated using Fisher’s exact test; the collection of p-values were subsequently adjusted using the FDR procedure [22].

## Results

### Selection of High and Low haloperidol-responsive lines

**Figure 1:**
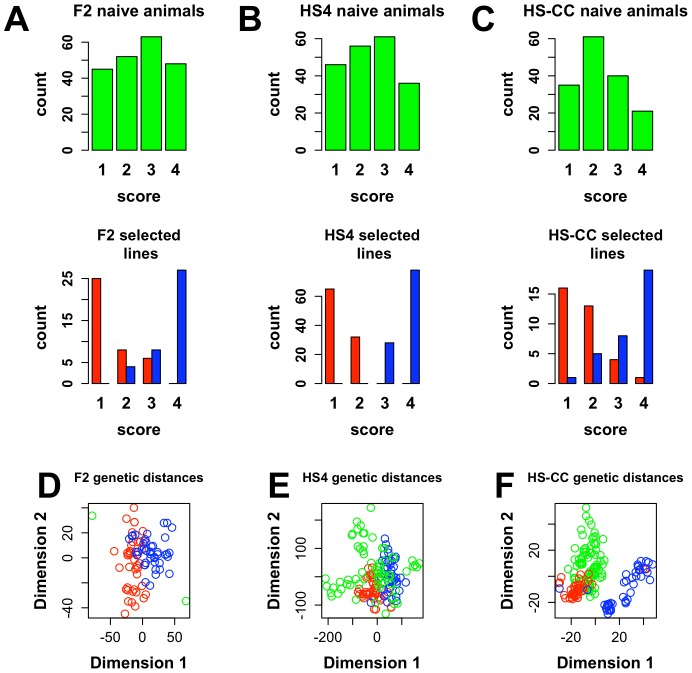
Phenotypic and genetic differences between naïve animals and selected lines. “Green”, naïve animals; blue, High line; red, Low line. A–C: Top, distribution of catalepsy responses in the naïve populations—high scores denote responders. Bottom, distribution of catalepsy responses in the selected lines. The three selected populations display differences in scores, showing successful selection. D–F: Multidimensional scaling of the genetic distances between individuals. The selected populations appear distinct from each other and closer together due to allele loss/fixation.

Procedures for testing and selecting mice have been described previously [1], [4]. Briefly, equal numbers of males and females (∼200 total) from the founder populations (F_2_, HS4 and HS-CC) were phenotyped for haloperidol response using a two-step process that resulted in assigning animals to one of four groups; “1” was the least responsive, and “4” was the most responsive. Breeding pairs were selected from the most extreme response groups. The selection and breeding were continued for two additional generations; in the third generation, parents were bred for three rounds to produce progeny for gene expression. As illustrated in Figures 1 (A–C), all three selections produced significant phenotypic segregation.

### Genome-wide differences between selected lines

The F_2_ and HS4 founders and their respective selected lines were genotyped using a SNP panel described elsewhere [15]. A denser genotyping platform was used for the HS-CC (see Methods). Each genome was encoded as a long vector with entries corresponding to allelic content, allowing computation of genome-wide genetic pairwise distances between samples. Genetic analysis used the Analysis of Molecular Variance (AMOVA) approach [17]; see Methods. As illustrated in Figure 1 D–F, pairwise distance variability was higher in the founder animals compared with the selected lines, reflecting allele fixation and loss of genetic variability. Additionally, the individual samples from the selected lines clustered together, indicating that selection results in two genetically distinct populations (AMOVA F-test p<10^-5^).

### Gene-expression differences between selected lines

Differential striatal gene expression for the three pairs of selected lines was calculated using the eBayes modified t-statistic available in Bioconductor (www.bioconductor.org) and further adjusted using the False Discovery Rate (FDR) [23]. Among the high variability transcripts used in the analysis, the number of differentially expressed transcripts (FDR<0.1) was significantly higher in the HS-CC compared with the F_2_ and HS4 selections (445 versus 113 and 33, respectively). There were no differentially expressed transcripts common to all three selections. The gene transcripts showing a significant differential effect are found in Dataset S1, in File S1.

### Construction, validation and annotation of the coexpression networks

**Figure 2:**
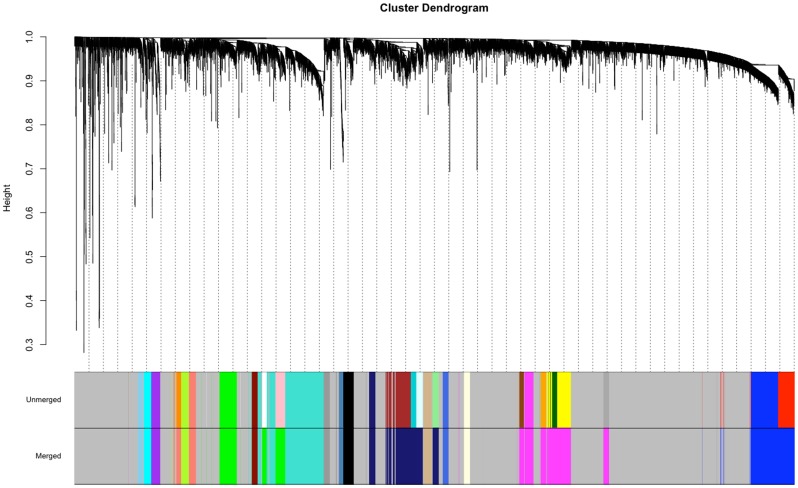
Hierarchical clustering of gene modules and module color assignments. Top: clustering tree. Bottom: initial unmerged colors and subsequent merged (final) module color assignments.

We constructed gene coexpression networks following the WGCNA procedure [13], [18]. We denoted as the network the collection of all transcripts (network nodes) together with the connection strength between them (edge weights). Transcript expression levels were correlated across samples, giving an initial estimate of the network adjacency; subsequently, this value was raised at a power β, resulting in a more robust approximation of the connection strength between transcripts [18]. In the current study, β =  10 was used. For a node, network connectivity is defined as the sum of all its network connection strengths; an approximately exponential distribution of node connectivity defines a scale-free network structure [24]. Intramodular connectivity is related but distinct from network connectivity: for each transcript the summation of connection strengths considers only connections within a module. To facilitate an unbiased comparison of network structure, we constructed a “consensus” network using 6755 gene transcripts with high detection levels in all three datasets (see Methods); the adjacency in this network was the average adjacency across the three selections. Clustering the network structure, followed by merging of modules with close eigengenes, revealed 25 modules in the consensus network, identified by arbitrary colors (Figure 2). Transcripts not assigned to any cluster were denoted as “grey”. Using a bootstrapping procedure, it was verified that all modules had an average adjacency significantly higher than what could be detected by chance. The module sizes varied between 40 and 471 transcripts. Gene Ontology (GO) annotation of the consensus modules revealed association with distinct biological processes (Dataset S2, in File S1). Two of the modules (“Grey60” and “Pink”) were both enriched in gene transcripts associated with locomotor behavior. To further explore the biological significance of the coexpression modules, we cross-referenced the module membership with known markers of distinct cell types in the murine brain [25]. Four modules (“Darkred”, “Midnightblue”, “Pink” and “Turquoise”) were enriched with transcripts associated with neuronal cell types, one module (“Black”) with oligodendrocytes markers and two modules (“Purple” and “Tan”) with astrocyte markers (Bonferroni corrected p<0.05). Modules were also examined for overrepresentation of transcription factor binding sites (TFBS); the probability of enrichment was assessed using Fisher’s exact test followed by an FDR adjustment. At FDR<0.1, we identified 8 modules enriched with specific TFBS (Dataset S3, in File S1). Because pairs of TFBS often contribute to gene expression in cooperative fashion [26], [27], [28], [29], we also searched for modules with overrepresented TFBS pairs. Eleven such modules were found (Dataset S4, in File S1). Because of the degeneracy of TFBS, there are multiple transcription factors (TFs) that can compete for TFBS; however, even taking this fairly broad view of TFs, we were unable to align differential TF expression or differential TF connectivity (see below) with selection-induced changes in network structure.

### Coexpression differences associated with selection

We evaluated whether selection induced significant change or disruption in coexpression structure, using the consensus modules as a common baseline. For each selection, two separate networks were constructed, using the Low and High samples respectively; examination of the intramodular connectivity values revealed that many transcripts changed connectivity. To evaluate the statistical significance of the observed differences, a set of networks (N = 200) was constructed using a mixture of High and Low samples; differences in connectivity values were used to construct an empirical distribution of connectivity changes that can occur by chance. Differences between the High and Low networks were normalized against the empirical distribution. Using this procedure, each transcript connectivity change between High and Low networks was assigned a Z score; scores Z>2 were considered significant. We found a relatively large number of transcripts significantly changed network connectivity: 458 (7.0%), 499 (7.6%) and 1537 (23.4%) in F_2_, HS4 and HS-CC populations, respectively. However, as for differential expression, none of the differentially connected transcripts were in common across the three selections.

We also evaluated the collective, cumulative change in connectivity of all transcripts within a module. As detailed in Methods, we used an adapted version of the module preservation procedure that is part of the WGCNA pipeline [14]. For each module, intramodular connectivity and other network statistics values (see Supplementary Methods, in File S2) were arranged in a vector. Module-wide connectivity differences were evaluated by examining the correlation values between the vectors corresponding to the High and Low networks. Our analysis revealed that, for each selection, several modules significantly (Z<-2) changed intramodular connectivity structure: 4 modules in F_2_, 12 in HS4 and 21 in HS-CC; a complete listing of changes in network statistics is available in Dataset S5, in File S1. As in differential expression and transcript-level differential connectivity, the changes in HS-CC were more pronounced. Importantly, three modules changed significantly in all three selections: “Green”, “Grey60” and “Pink” modules; the Z scores for these modules are listed in Table 1.

**Table 1:** List of disruption Z scores.

Module/Cross	cor.kIM	cor.kME	cor.kMEall	cor.ADJ	cor.clusterCoeff	cor.MAR
Green/F2	−2.15	−1.71	−1.42	−2.43	−1.09	−1.15
Grey60/F2	−2.51	−3.08	−0.85	−4.28	−1.53	−1.43
Pink/F2	−2.09	−1.75	−2.35	−1.98	−1.6	−1.32
Green/HS4	−2.19	−1.87	−0.58	−2.6	−2.26	−3.15
Grey60/HS4	−2.09	−1.53	−0.88	−1.98	−1.15	−1.58
Pink/HS4	−2.73	−1.62	−0.59	−1.89	−0.2	−1.23
Green/HS-CC	−2.98	−1.32	−1.16	−6.83	−0.92	−1.25
Grey60/HS-CC	−2.47	−1.36	−1.08	−6.23	−1.18	−0.61
Pink/HS-CC	−3.95	−4.07	−3.05	−5.08	−2.61	−2.87^1^

1 Three modules (Green, Grey60 and Pink) displayed significant disruption (cor.kIM absolute value z scores above 2) in all three datasets.

### Integration of genotype and coexpression in the HS-CC data

**Figure 3:**
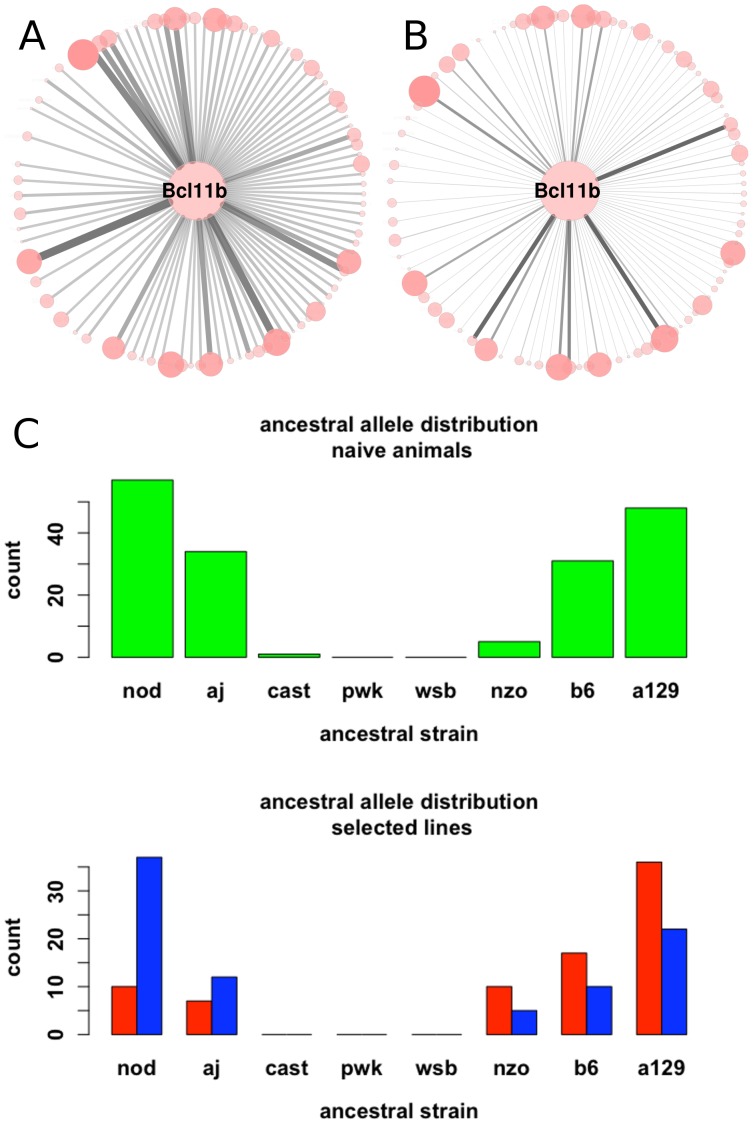
*Bcl11b* connectivity and allelic differences between High and Low selected lines – HS-CC founders. A: *Bcl11b* connectivity patterns in the High network. For visual clarity, only edges involving *Bcl11b* are represented. Edge thickness and opacity are proportional with the edge weight (adjacency). Node size (except *Bcl11b*) is proportional with modular connectivity. B: Low network *Bcl11b* connectivity pattern. C: Allele distribution for *Bcl11b* in the naïve HS-CC animals (“Green”, top) and in the High and Low selected lines (red and blue, bottom). NOD and A/J alleles are more prevalent in the High group (blue) while NZO, B6 and A129 are more prevalent in the Low group (red). Strains: C57BL/6J (B6); A/J (A); 129S1/SvImJ (129); NOD/LtJ (NOD); NZO/HILtJ (NZO). CAST/EiJ (CAST). PWK/PhJ (PWK), WSB/EiJ (WSB).

The dense genotyping of the HS-CC samples facilitated the integration of genetic and coexpression data. We identified genomic regions with significant allele distribution differences between the High and Low selected lines. By combining the genotype data from the founder inbred lines, it was possible to infer, for each animal, the most probable ancestral alleles for each genomic interval [21]. The allele distribution was then arranged in a contingency table with rows corresponding to the two selections and columns corresponding to the ancient strain allele origin. A p-value of significant differences was computed using Fisher’s exact test; the p-values were then adjusted using the FDR procedure [22]. This procedure revealed the genomic intervals with ancestry segregating during selection (FDR<0.1); 2047 out of the 6755 network transcripts were located within these intervals. The transcripts of highest interest 1) fell within the three modules of interest, 2) displayed significant module connectivity changes and 3) were located within segregating genomic intervals. The identity of these genes is presented in Table 2. We consider that these transcripts offer the best evidence of direct, functional links between selection and phenotype and the molecular level. A number of these genes have previous association with similar phenotypes. In the “Grey60” module, *Myo5a,* falls within the range of a previously detected QTL for haloperidol induced catalepsy [30]. Also in the “Grey60” module, *Rps6ka5* has been previously associated with dopamine processing within striatal neurons [31]. In the “Green” module, *Spock1* has been previously associated with genetic effects on tyrosine hydroxylase levels within dopaminergic neurons [32]. One of the “Pink” module gene transcripts with significant allelic and connectivity changes, *Bcl11b*, has been previously shown to strongly influence striatal gene expression [33] and transcriptional dysregulation in Huntington’s disease [34]. We selected *Bcl11b* to illustrate connectivity and allelic differences in Figure 3.

**Table 2:** Genes with connectivity and allele origin differences.

Symbol/Module	High Network Module Connectivity Rank	Low Network Module Connectivity Rank	zScore Connectivity Change	Raw p value allele differences	FDR value allele differences
Arf3/Grey60	52	31	-2.7	8.95E-05	0.0004
Myo5a/Grey60	33	47	2.3	5.80E-17	1.08E-14
Rps6ka5/Grey60	10	36	4.9	0.0001	0.0007
Cyp2a5/Green	115	165	2.2	0.002	0.008
Hist1h4f/Green	15	127	2.6	0.0009	0.003
Nckap1l/Green	52	29	-2.002	2.60E-07	3.05E-06
Pex26/Green	119	103	-2.02	1.65E-05	0.0001
Rab40c/Green	73	16	-2.2	0.0003	0.001
Spock1/Green	116	36	-4.1	0.001	0.003
2300002D11Rik/Pink	38	71	2.4	4.74E-05	0.0002
A130092J06Rik/Pink	75	36	-2.001	0.003	0.009
Bcl11b/Pink	24	6	-2.2	6.66E-05	0.0003
Kcnab1/Pink	37	11	-2.9	3.94E-06	3.04E-05
Pde1b/Pink	2	2	-2.04	2.60E-07	3.05E-06
Pou3f1/Pink	30	83	2.6	8.63E-05	0.0004
Prosapip1/Pink	43	10	-2.5	1.27E-10	4.67E-09
Tbc1d10c/Pink	90	66	-2.02	0.002	0.008
Tmem90a/Pink	77	29	-2.4	3.79E-08	5.87E-07^2^

2 For the three modules affected by selection, a number of genes change connectivity significantly, as indicated by change in connectivity rank and z Score. The same genes fall within genomic regions that segregate between High and Low populations.

## Discussion

Haloperidol-induced catalepsy is an ideal phenotype for integrative analysis of gene networks, genetic diversity and behavior. The mechanism of drug action is well established (blockade of D_2_-like dopamine receptors), the striatum is the major target for the extra-pyramidal response and the phenotype is highly heritable [2], [3]. Differences in response among inbred mouse strains are not due to differences in D_2_ receptor density, the relative amounts of the long and short receptor isoforms or haloperidol pharmacokinetics [3], [35], [36], [37]. The genetic differences are not haloperidol specific; for example, the D_2_–D_3_ receptor antagonist, raclopride, shows the same inbred strain distribution pattern [2]; all of the selected lines differed significantly in their response to raclopride (data not shown). However, the genetic differences are not paralleled by differences in response to D_1_ receptor antagonists, e.g., SKF 23390 [37].

From a translational perspective, comparison of mouse populations with different genetic backgrounds is an essential first step. Before any genetic or molecular mechanisms can generalize to humans, they must be shown to be similar in genetically distinct subpopulations of the same species. Broadly speaking, a systems-biology approach was used to detect mechanisms that transcend a specific genetic background. Key to this approach was the inclusion of a selection from the genetically diverse HS-CC founders. As noted by [5] and confirmed by genome-wide next-generation sequencing [38], F_2_ intercrosses and four-way and eight-way HS populations formed from standard mouse laboratory strains will capture only a fraction of *Mus musculus* genetic diversity; F_2_ and standard HS populations are actually more similar than different. In contrast, the eight strains used to form the HS-CC, which includes three wild-derived strains, capture approximately 90% of the available allelic diversity. However, despite the marked difference in diversity, the basal HS-CC striatal coexpression network is similar albeit not identical to that found in F_2_ and HS4 animals [13].

The WGCNA is one of several graph/network approaches for analyzing gene coexpression structure. We recognize that other approaches and indeed subtle variations in the implementation of the WGCNA can yield different results [4], [39]. The WGCNA aligns strongly with two principles. The first is that coexpressed genes are likely to share biological functions; importantly, this principle allows putative annotation of genes and noncoding RNAs with no known function. In our case, we used an unsigned network [6], [18], which implies that both positively and negatively correlated genes are coexpressed and often assigned to the same module. The second principle is that the network structure is scale-free and follows a power law distribution; this implies that the network is best described by a few highly connected hub genes, with most of the nodes sparsely connected [6]. WGCNA was used to show that it was at the level of network structure and not differential expression that one could discriminate the nonhuman primate and human brain transcriptome and the regional brain differences in the human transcriptome [40], [41]. Importantly from a translational prospective, recent work has demonstrated that eQTL in combination with coexpression network analysis can identify novel candidate genes related to schizophrenia [42], [43]. The WGCNA has been used to dissect the transcriptome into modules associated with specific cell types (neurons, oligodendrocytes, astrocytes and microglia), specific organelles and synaptic functions [41]; our data replicated this observation. Recent work [44] has also illustrated that the WGCNA is sufficiently sensitive to detect behaviorally relevant differences in network structure even among an inbred strain.

Data previously reported [4] were the first to show that selection for a behavioral phenotype had marked effects on WGCNA generated network structure. In this example, the HS4 was specifically created to assess selection for haloperidol response; of the founder strains, two (D2 and C) are haloperidol responsive and two (B6 and LP) are non-responsive [37]. For the gene coexpression analysis of the HS4 selected lines, the construction of the consensus network from the responsive and non-responsive lines was key; the data illustrated that the module showing the most significant effects of selection was one enriched in genes associated with intracellular signaling and locomotor behavior (similar to the “Pink” module of the current study). The consensus network used in the current study was somewhat different from that reported [4], in that a larger number of transcripts were included in the analysis and data were collapsed across three different selections. An important observation [4] was that selection had only modest effects on differential gene expression. This point was extended in the current study and further it was found that there was no overlap in differentially expressed genes among the three selections. These data align with previous observations that haloperidol-response quantitative trait loci (QTL) are genotype dependent [1], [3], [30], [45], [46]. The consensus network approach revealed that selection for haloperidol response, regardless of genetic background, disrupted similar aspects of network structure; the key disruptive features were changes in intramodular connectivity. Three modules (“Grey60”, “Pink” and “Green”) were consistently affected. But this commonality was only detectable at the module level; connectivity changes among individual transcripts were founder population unique. Thus, within the striatal transcriptome the common selection element is the gene module and not individual gene connectivity. It is of interest to note that the specificity of the analysis was largely generated by the F_2_ data; the more complex crosses recruited additional modules such that in the HS-CC sample, nearly all of the modules within the striatal gene network were affected.

The question arises as to whether or not the apparent relationship between genetic diversity and the extent of module involvement is a general rule: does selection recruit a more complex biology in the more complex crosses? The data presented here cannot address this issue; replicate selections from the HS4 and HS-CC populations would be needed to eliminate the effects due to random allele fixation.

The module-centric/genotype-dependent view of selection, heritability and behavior could be generalized to other phenotypes and other populations under certain conditions. While publically available and behaviorally relevant datasets are generally too small to accurately construct gene networks, a meta-analysis approach [44] could address this issue provided the behavior is measured under similar conditions. It has been argued [47] that it is gene-environmental interactions that are key to understanding the natural variation in behavior, which is the basis of selective breeding. Our data provide an example of holding the environment “constant”, varying genetic background and observing selection-induced changes in network structure. But in real-world situations, e.g., a genome wide association study of natural variation, neither the background nor the genotype is held constant. Thus, the possibility of aligning phenotypic variation with connectivity becomes more difficult.

Given this complexity and the fact that module(s) specifically and networks generally may be the key to understanding heritability, how best to investigate the gene-behavior relationship, especially with a translational perspective? Here, we simply offer one approach. Some behaviors, especially those that substantially involve subcortical circuitry, have a large number of relevant isomorphic animal models. With these models in hand, it is possible to use network techniques to detect key modules; the translational goal is to determine which genes within the key module(s) are targets for manipulation and behavioral modification. We argue that the inclusion of the HS-CC or a related population such as the Diversity Outcross [48] is key to the translational perspective. The genomic granularity and allelic diversity allowed a reasonably accurate alignment of changes in connectivity with specific genomic regions that segregate with selection (Table 2). In addition and only in the HS-CC population, there were eight transcripts with significant differential expression and significant changes in connectivity in the key catalepsy modules (Dataset S6, in File S1). This process significantly reduced the number of targets identified for manipulating haloperidol response. By definition (because they are members of a key coexpression module), they have a role in haloperidol response.

Another target driven approach is to focus on the “hub” genes. From the scale-free network perspective, hub disruption will have the greatest affect on modular connectivity. It is not necessary that the hub targets be genes that are affected by selection although focusing on these genes provides insight into the causes of natural variation. In the three selection consensus modules, there are a number of hub genes previously shown to significantly affect locomotor behavior and in some cases haloperidol response. In addition to *Drd2* (“Pink”), these include *Rgs9, Pde10a* and *Chat* (all “Pink”), *Tcf4* (“Green”) and *Lrrk2* and *Foxp2* (“Grey 60”) [49], [50], [51], [52], [53], [54], [55]. It is also of interest to note that two histone demethylases (*Jmjd1a* and *Jmjd2b*) are key hub genes (“Grey60” and “Pink”, respectively), perhaps suggesting an epigenetic strategy for modifying haloperidol response.

Overall, the data presented here argue that a systems biology approach is needed, at least in some contexts, to investigate the relationships between genes and behavior. The results from the three independent selections illustrated that at the gene transcript level, neither differential expression, nor differential connectivity in one selection population predicted similar results in another selection population; however at the module level, connectivity changes did overlap. Extracting module-dependent information requires relatively large gene expression datasets, which in turn facilitate an accurate assessment of the covariance structure. This assessment can be improved by substituting RNA-Seq for microarray based strategies [56].

## Supporting Information

File S1 Contains: Dataset1: Gene transcripts showing a significant differential effect. Dataset2: Gene Ontology (GO) annotation of the consensus modules. Dataset3: Transcription factor binding sites (TFBS) enrichment of consensus modules. Dataset4: Transcription factor binding site pair enrichment of consensus modules. Dataset5: Network changes quantified using Z scores. Dataset6: Gene transcripts with significant changes in both expression and connectivity.
(ZIP)

File S2 Supplementary Methods: Definition of network statistics used in the analysis.
(DOCX)
