# Supplementary material for: Differential Network Analysis Reveals Genetic Effects on Catalepsy Modules
Source: PLoS One. 2013 Mar 21;8(3):e58951. doi: 10.1371/journal.pone.0058951 (PMC3605410; doi:10.1371/journal.pone.0058951)
Supplement: File S2 — Supplementary Methods: Definition of network statistics used in the analysis. (DOCX) [file pone.0058951.s002.docx]

# Supplementary Methods

## Evaluation of Network Changes

In many biological network applications, it is of interest to evaluate to what extent network properties are preserved or disrupted across biological conditions. Recent work (Langfelder et al., 2011) has introduced a comprehensive and powerful method for evaluation of preservation of network properties. For any collection of network nodes of interest (module), preservation statistics are created by comparing network/module statistics against similar values compiled from randomly selected groups of nodes. Broadly speaking, bootstrapping or random selection is performed over network nodes.

In the current application, the goal is to detect significant changes in network structure. Even though modules might be highly preserved across biological conditions, this does not preclude the emergence of subtle changes in network structure that are not enough to render the module non-preserved, but nevertheless are statistically significant and, potentially, biologically meaningful. To detect these changes, we adapted the module preservation procedures outlined in (Langfelder et al., 2011). In essence, we create separate networks corresponding to the different biological conditions; differences between these networks are evaluated against changes that could occur by chance. An empirical distribution of random changes is compiled by constructing networks using a mixture of samples from both biological conditions. Bootstrapping is performed over samples as opposed to network nodes.

To facilitate a clear comparison of network properties, we first constructed a consensus network using average correlation values from the three datasets corresponding to three different genetic backgrounds. Clustering the consensus adjacency matrix resulted in the detection of a set of consensus modules; this consensus module assignment was used for evaluation of network preservation and disruption.

In this section we review the module preservation statistics introduced in (Langfelder et al., 2011), we describe our modifications, and we detail the module disruption values detected in our data.

## Module preservation statistics

(Langfelder et al., 2011) outline several different types of network statistics; in the current study we use density based and connectivity based statistics. Density based statistics determine whether module nodes remain highly connected across networks. Connectivity statistics evaluate whether the connectivity patterns between nodes remains preserved across networks. Preservation statistics make use of more general network concept definitions. For complete definitions of network concepts and preservation measures the reader is referred to (Langfelder et al., 2011), Equations 1-20.

Node adjacency: $a_{ij}=cor(i,j)$ or the Pearson correlation of expression values over samples

Adjacency matrix $A=(a_{ij})$ n by n matrix of pairwise adjacencies

$vectorizeMatrixA=(a_{21}...a_{n1}...a_{n-1,n})\text{ }$ a listing of non-redundant adjacency values

Node connectivity $k_{i}=\sum_{j\neq i} a_{ij}$sum of adjacencies of a node

Intramodular node connectivity $kIM_{i}^{(q)}=\sum_{\begin{matrix} i\in Q \\ i\neq j \end{matrix}} a_{ij}^{(q)}$ sum of connection strengths of a node restricted within the containing module

Module eigengene connectivity $kME_{i}^{(q)}=cor(x_{i},E^{(q)})$, where $x_{i}$ is the profile of node i and E is the eigengene of module q

Clustering coefficient $clusterCoeff=\frac{\sum_{j\neq i} \sum_{m\neq j,i} a_{ij}a_{jm}a_{mi}}{(\sum_{j\neq i} a_{ij})^{2}-\sum_{j\neq i} a_{ij}^{2}}$

Maximum adjacency ratio $MAR_{i}=\frac{\sum_{j\neq i} a_{ij}^{2}}{\sum_{j\neq i} a_{ij}}$

The network statistics defined above apply to an individual node $i$; for a given module $q$, the collection of network statistics can be organized in a vector. To evaluate preservation of a network statistic in a module, vectors originating from two different networks are correlated; high correlation values correspond to strong preservation. Furthermore, statistical significance of preservation values can be obtained by using a Z score (Langfelder et al., 2011):

where *a* indicates a network statistic, and μ, σ are the mean and standard deviations of the module measurements generated using the bootstrapping procedure. The essential difference between our disruption Z score and the preservation Z scores defined by Langfelder et al., (2011) is the way in which μ and σ are computed. For preservation Z scores, μ and σ are computed using random groups of nodes. For disruption Z scores, μ and σ are computed from comparing two networks that are constructed using a mixture of samples from the two biological conditions.

In many cases, real module measurements are higher than measurements for random groups; Z values between 2 and 10 denote moderate preservation while Z values higher than 10 indicate strong preservation.

## Module disruption statistics

We modified the procedure outlined above to detect statistically significant differences between two networks that use the same module assignment. All the network measurement definitions are identical to (Langfelder et al., 2011), Equations 1-20. However, the manner in which disruption Z scores are computed is changed. Networks 1, 2 are constructed using samples that belong to two different groups. Network preservation statistics are computed between these two networks.

A collection of networks is constructed using a random mixture of samples from the two categories; preservation statistics between pairs of these networks are collected; next, mean and standard deviation$\mu_{a}^{mix},\text{ }\sigma_{a}^{mix}$ are computed. Finally, disruption Z scores are computed based on these quantities.

We note that generally one can expect Z preservation scores to be positive because preservation is higher for modules compared with random groups of nodes. However, Z disruption scores are often negative because preservation is lower between Network1 and Network2 (samples belong to distinct categories) compared with networks constructed using mixture of samples; the latter do not have a biological basis for changes in network structure. Following (Langfelder et al., 2011), we denote Z disruption scores between 0 and -2 to be moderately disrupted while Z scores lower than -2 are considered strongly disrupted.

Langfelder, P., Luo, R., Oldham, M.C., and Horvath, S. (2011). Is my network module preserved and reproducible? *PLoS Comput Biol* 7**,** e1001057.
